# Evaluating Landscape Options for Corridor Restoration between Giant Panda Reserves

**DOI:** 10.1371/journal.pone.0105086

**Published:** 2014-08-18

**Authors:** Fang Wang, William J. McShea, Dajun Wang, Sheng Li, Qing Zhao, Hao Wang, Zhi Lu

**Affiliations:** 1 Smithsonian Conservation Biology Institute, National Zoological Park, Front Royal, Virginia, United States of America; 2 School of Life Sciences, Peking University, Beijing, China; 3 Department of Fish, Wildlife, and Conservation Biology, Colorado State University, Fort Collins, Colorado, United States of America; SUNY College of Environmental Science and Forestry, United States of America

## Abstract

The establishment of corridors can offset the negative effects of habitat fragmentation by connecting isolated habitat patches. However, the practical value of corridor planning is minimal if corridor identification is not based on reliable quantitative information about species-environment relationships. An example of this need for quantitative information is planning for giant panda conservation. Although the species has been the focus of intense conservation efforts for decades, most corridor projects remain hypothetical due to the lack of reliable quantitative researches at an appropriate spatial scale. In this paper, we evaluated a framework for giant panda forest corridor planning. We linked our field survey data with satellite imagery, and conducted species occupancy modelling to examine the habitat use of giant panda within the potential corridor area. We then conducted least-cost and circuit models to identify potential paths of dispersal across the landscape, and compared the predicted cost under current conditions and alternative conservation management options considered during corridor planning. We found that due to giant panda's association with areas of low elevation and flat terrain, human infrastructures in the same area have resulted in corridor fragmentation. We then identified areas with high potential to function as movement corridors, and our analysis of alternative conservation scenarios showed that both forest/bamboo restoration and automobile tunnel construction would significantly improve the effectiveness of corridor, while residence relocation would not significantly improve corridor effectiveness in comparison with the current condition. The framework has general value in any conservation activities that anticipate improving habitat connectivity in human modified landscapes. Specifically, our study suggested that, in this landscape, automobile tunnels are the best means to remove current barriers to giant panda movements caused by anthropogenic interferences.

## Introduction

Habitat loss and fragmentation are considered the primary threats to many endangered species [1]. Movement corridors can mitigate the negative effects of habitat fragmentation by connecting isolated populations [2]. Although corridor establishment and restoration are commonly proposed to compensate for the negative effects of habitat fragmentation, managers typically neglect habitat selection and migratory behavior of focal species when identifying planned location of corridors [3]. Common criticisms of corridor studies include the use of geographic metrics (e.g. slope, elevation, etc.) to measure the landscape connectivity without considering more animal-centric measures [4], or a reliance on expert opinion that makes validation and applicability to other regions problematic [5]. Validated models based on empirical data of animal habitat use should be used for predictive or planning purposes [4], [5].

Giant panda (*Ailuropoda melanoleuca*) is a large mammal that requires movement corridors for long-term conservation. This species is restricted to approximately 24 isolated populations, 13 of which are considered to be at a high risk of extinction [6]. While the core populations reside within established nature reserves with intensive protection efforts, marginal populations are still losing habitat as well as population numbers [7], [8]. Forest corridors have been identified and advocated for giant pandas since the 1980s, with the goal of maintaining or restoring the habitat connectivity between nature reserves [9]. However, few of the proposed corridors were found to be functional, as studies revealed that the trend of habitat fragmentation, as well as population differentiation, continued [7], [10]. Giant panda corridor studies either used expert opinion to determine the environment-species relationships [11], or constructed models based on coarse resolution landscape metrics at regional scale [8], [12], [13], both practices which may limit the practical application of their results at specific locations.

The purpose of this study was to use a key linkage area between two known giant panda populations to evaluate landscape options for corridor establishment. This area has been identified as one of the three most important potential corridors for giant panda movement in the Qinling Mountains [9], [14]. Our study aims to (1) model the habitat use of giant panda populations in a proposed corridor area; (2) predict potential giant panda movement pathways; and (3) evaluate alternative management strategies on corridor effectiveness.

## Materials and Methods

Field survey was approved by the Shaanxi Forestry Department (SFD).

### Study Area

**Figure 1:**
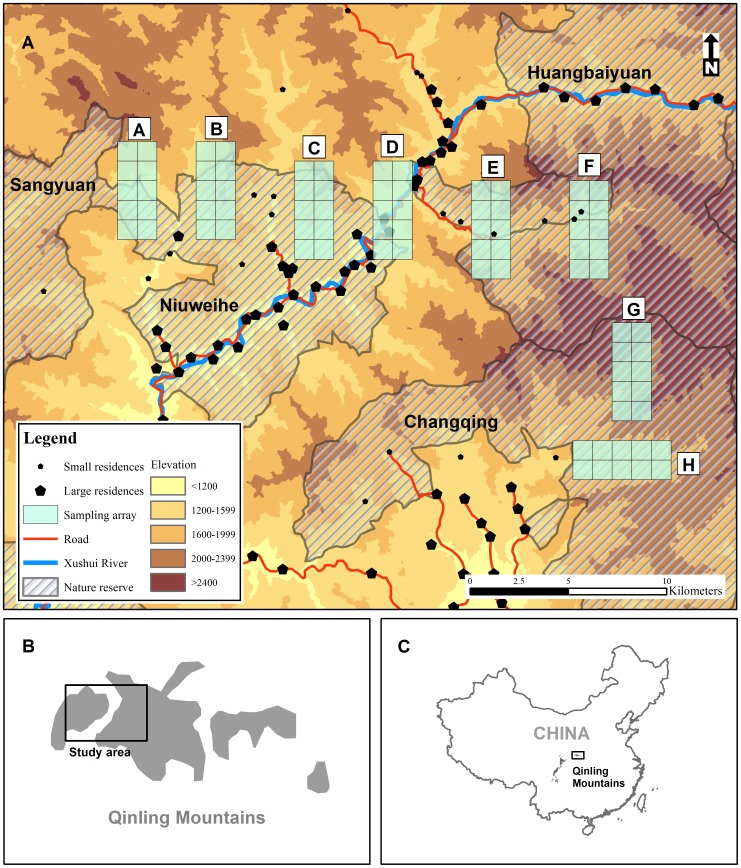
Sampling arrays layout in the proposed corridor area. Arrays A, B and C are located in Niuweihe Nature Reserve, arrays E, F, G, and H were located in Huangbaiyuan and Changqing Nature Reserves, and array D was located between nature reserves along the Xushui River Valley (A).The grey color in lower two figures indicates the known giant panda distribution. The study area was in the western region of Qinling Mountains (B), and located in the center of China (C).

Our study area is a 400 km^2^ region along the Xushui River Valley (XRV) in the western Qinling Mountains, which covers three nature reserves and a key linage area between giant panda populations (Fig. 1A). Huangbaiyuan, Changqing, and eastern Niuweihe Nature Reserves are located to the east of the XRV, and harbor a population of approximate 60 giant pandas [14]. Western Niuweihe Nature Reserve, located to the west of the valley, has an estimated population of approximate 20 giant pandas [14]. Previous studies suggest that there may be dispersal movements of giant pandas between the Huangbaiyuan-Changqing population and the Niuweihe population through the XRV area [15].

The XRV is a valley corridor, where complex topography and human infrastructures (e.g., residences, agriculture practice, road, etc.) along a river valley might be barriers for the connectivity of animal populations (Fig. 1A). The proposed XRV Corridor is comprised of four dispersed communities and numerous residences along a two-lane highway (Yangxian-Taibai Highway) that bisects the valley along its north-south axis [16]. The shortest distance between the known western and eastern giant panda populations is approximate 16 km based on previous surveys (Fig. 1B) [14].

### Sampling design and Data Collection

We conducted giant panda surveys using passive infrared cameras (TCB-100, Huangwu Inc., Wuhan, CHINA; Reconyx PC800, Reconyx, Wisconsin, USA). In addition, we used a sign plot method to detect evidence of giant panda activity (feces, tracks, and feeding signs) as supplementary information for the modeling process (see below). The survey season ran from April 2010 through March 2013 (35 months). We created eight 10 km^2^ sampling arrays along the east-west axis of the XRV. Four arrays to the western side of the XRV were located in Huangbaiyuan and Changqing Nature Reserve, three eastern arrays were located in Niuweihe Nature Reserve, and one array was located within the XRV. Each 10 km^2^ sampling array was divided into ten 1×1 km grid cells (Fig. 1). At the beginning of each sampling period, we randomly selected three grid cells in each array and placed one survey site in each selected grid cell. Field staff were allowed to select the most probable location to detect giant panda within each grid cell with the limitation of no concurrent sample locations being placed within 500 m.

Cameras were mounted on trees 40 cm above the ground and operated 24 h per day with a 20 s delay between sequential photographs. For most camera set-ups, a few drops of scent lure (Carman's Magna-Glan Lure, Sterling Fur, Ohio, USA) was applied 1–2 meters in front of the cameras to slow animal movement and to compensate for slow camera trigger times. At the end of each survey period (30–50 days, mean 38 days), the digital flash cards were collected, and the cameras were moved to the next survey locations.

A 40 m×20 m plot was surveyed for panda signs at each camera location. Field staff set up a plot centering on each camera location, and recorded all evidence of giant panda activity within the plot. Surveys were conducted before and after camera period at each location. We combined the data with camera trap data detections to generate a giant panda distribution layer, and used the layer for corridor modelling (least-cost model and circuit model).

For occupancy modelling, we reviewed previous giant panda habitat studies [17]–[20] and identified 13 potentially important occupancy covariates (Table 1). Geo-referenced human infrastructure data (i.e. roads, residences, and croplands) were collected using a handhold GPS unit (Garmin 60CSx, Garmin, Schaffhausen, Switzerland). We categorized residences into small (<3 households) or large (≥3 households), and roads into local (<2 lane) or provincial (≥2 lanes). We used a 30-m resolution DEM [21] to delineate elevation and slope. The SFD provided a nature reserve database with geo-referenced boundary and protected level (national, provincial, and non-nature reserves). The Taibai Forestry Company provided a forest database to categorize forest age as primary or secondary. We used 30 m Landsat data ETM+ satellite imagery (path 128, row 037, date 2008-07-06) to conduct a supervised classification of land cover across the study landscape [22], and aggregated forest composition into four classes: coniferous forest, broad-leaved forest, mixed forest and non-forest. The distribution of bamboo across the study area was surveyed by the SFD during 2007 to 2010 by conducting 413 100 m^2^ vegetation plots in and outside nature reserves (Table S2, i.e. coordinates, bamboo species, and elevation). We modeled the distribution of bamboo within the study area using Maxent [23]. Maxent is a machine learning program that estimates a species' probability distribution by finding the probability distribution of maximum entropy (i.e., that is most dispersed), subject to a set of environmental constraints based on incomplete information about the species' distribution [23]. We included biophysical covariates used 300 m resolution WorldClim data [24] and four bio-geographic co-variables (elevation, slope, aspect and solar radiation), and followed established protocols for their inclusion [25], [26]. For detection covariates at each camera survey site, we obtained monthly mean temperature from WorldClim Data and categorized it into low (<5°C), medium (5–15°C), or high (>15°C), indicated which deployments included use of scent lure to attract animals, and categorized camera view as open or limited-view based on vegetation density.

**Table 1:** Covariates collected for occupancy and detection probabilities.

Name	Description
**Occupancy covariates**	
Elevation	Numeric (m)
Slope	Categorical (<5°, 5–20°, >20°)
Forest age	Categorical (primary, secondary)
Forest composition	Categorical (broadleaf, mixed, conifer, non-forest)
Bamboo distribution	Categorical (presence, absence)
Type of nature reserve	Categorical (not reserve, provincial, national)
Distance to small road*	Numeric (m)
Distance to large road*	Numeric (m)
Distance to any road	Numeric (m)
Distance to small residences	Numeric (m)
Distance to large residences	Numeric (m)
Distance to any residences*	Numeric (m)
Distance to cropland*	Numeric (m)
**Detection covariates**	
Lure	Categorical (applied, not applied)
Temperature	Categorical (<5°C, 5–15°C, >15°C)
Camera view	Categorical (open view, limited view)

*Excluded from model selection due to collinearity.

GIS layers were standardized to the same spatial projection and resampled to a 100 m×100 m resolution, and the mean value of elevation and slope, dominate land cover type, and the distance from cell center to human infrastructure (i.e. residences, roads, and cropland) were calculated.

An independent sign transect dataset that was collected by the SFD was used to validate our occupancy model (Table S3). The SFD surveyed for giant panda sign along 47 transects in forested habitat (total distance: 54 km) during 2010 to 2012. The trails were divided into 250 m segments and segments were categorized as with (114) or without (102) giant panda sign, with the center point and attribute of each segment entered into our GIS.

## Data Analysis

Our analyses included three steps. First, we constructed species occupancy modeling [27], [28] to identify important occupancy covariates, and mapped habitat suitability with the resulting predictive equation. Second, we used the inverse of the habitat suitability map as a resistance surface and constructed least-cost [29] and circuit models [30] to predict the potential dispersal pathways for giant panda in this landscape. For the last step, we varied the model parameters being considered under different conservation scenarios and assessed their effectiveness.

### Occupancy Modelling

We divided the camera-trapping sampling period at each survey site into 5-day segments [31]. The giant panda detection history for a segment was considered as “present” if any detection was made during the 5 days, and “absent” otherwise. Prior to occupancy modelling, we examined the collinearity of variables and selected covariates that have a pairwise correlation <0.7 for later analyses. We then examined the potential non-linear relationship between occupancy probability and each occupancy covariate by constructing a series of single-variable models. For each occupancy covariate, we selected from among its original, log-transformed, and quadratic forms, and used the one with the smallest Akaike's Information Criteria or AIC [32] in later analyses. To select detection covariates we constructed a general occupancy model including all occupancy covariates and investigated all combinations of detection covariates. We selected the top observation model based on AIC values and only considered the optimal detection covariates in subsequent analyses for occupancy covariate selection [33]. All possible combinations of occupancy covariates were examined, and a model-averaging approach was applied to calculate the weight-averaged parameter values for all variables found in the top models, as indicated by delta AIC values ≤2 [32]. We then used the model-averaged parameter values to create a predictive equation for calculating occupancy probability across the entire study area. The predictive model was validated using the SFD sign transect dataset. Giant panda was considered as “present” if giant panda signs were recorded, and “absent” if no panda sign was recorded at the location or any transect within 1 km of that location. Based on this dataset, we constructed receiver operating curves (ROC) for our habitat suitability map and calculated area under ROC (AUC). All occupancy modelling was conducted using the “unmarked” package [34] in R.

### Mapping Movement Pathways

We combined camera-trapping detections and sign plot records into panda presence locations. We generated the inverse of the predicted habitat suitability map as a cost surface [3] using ArcToolbox in ArcGIS 10.2 [35]. Based on this cost surface we constructed least-cost and circuit models to identify potential movement pathways. For least-cost modelling, we generated an accumulative cost surface layer and a path direction layer, and predicted least-cost-paths linking the source population and sink population using ArcToolbox in ArcGIS 10.2 [35]. In addition to the single optimal least-cost corridor, we selected five locations at each side of the river valley where giant panda were detected, and generated another 24 alternative pathways linking each possible set of points. To quantify the predicted pathways, we generated an index of movement cost per 100 meters using ArcToolbox, and calculated the mean movement cost for each pathway.

In addition to the line pathways predicted by least-cost model, we used the circuit modelling software Circuitscape 3.5 [30], and followed the method of Koen et al. [36] to model landscape connectivity for the entire XRV Corridor area.

### Scenario Analysis

To compare the effectiveness of alternative restoration options, we created hypothetical landscapes and compared their movement cost to the current landscape. The scenario settings were derived from our discussion with the local forestry department and nature reserves, as well as management options presented in recent government plans [14]. The three most possible management scenarios were: (1) forest/bamboo restoration, which is widely believed to increase giant panda habitat quality due to giant panda's association with mature forest [37]; (2) extensive residence relocation, which was commonly referred to “eco-migrant”, and has been conducted in numerous locations [38], [39]; and (3) automobile tunnel construction, whose primary purpose in the regions is to increase transportation speeds for commercial trucks and buses [40].

For the forest/bamboo restoration scenario, we assumed with proper forest management, the young successional forest in XRV area can be restored to mature forest with a bamboo understory. For the residence relocation scenario, we assumed the 7 large residences along the Yangxian-Taibai highway were relocated. For the automobile tunnel scenario, we assumed a 1 km section of current winding road be replaced by an automobile tunnel, and the abandoned road serve as a wildlife overpass. We recalculated the movement cost surface for each scenario (current situation plus three options), and compared the mean cost of the 25 potential pathways identified via the cost analysis process using a two-way Analysis of Variance (ANOVA), followed by a paired t-test. Holm's method was applied in the paired t-test to guarantee that the family-wise type I error probability was less than 0.05.

## Results

From 2010 to 2013, we surveyed 243 plot locations with a total effort of 9136 camera nights, 421 sign surveys, and 243 vegetation surveys (Table S1). Giant pandas were detected at 43 locations by camera traps and 133 locations by sign transects (Table S4).

### Giant Panda Occupancy Model and Habitat Suitability Mapping

**Figure 2:**
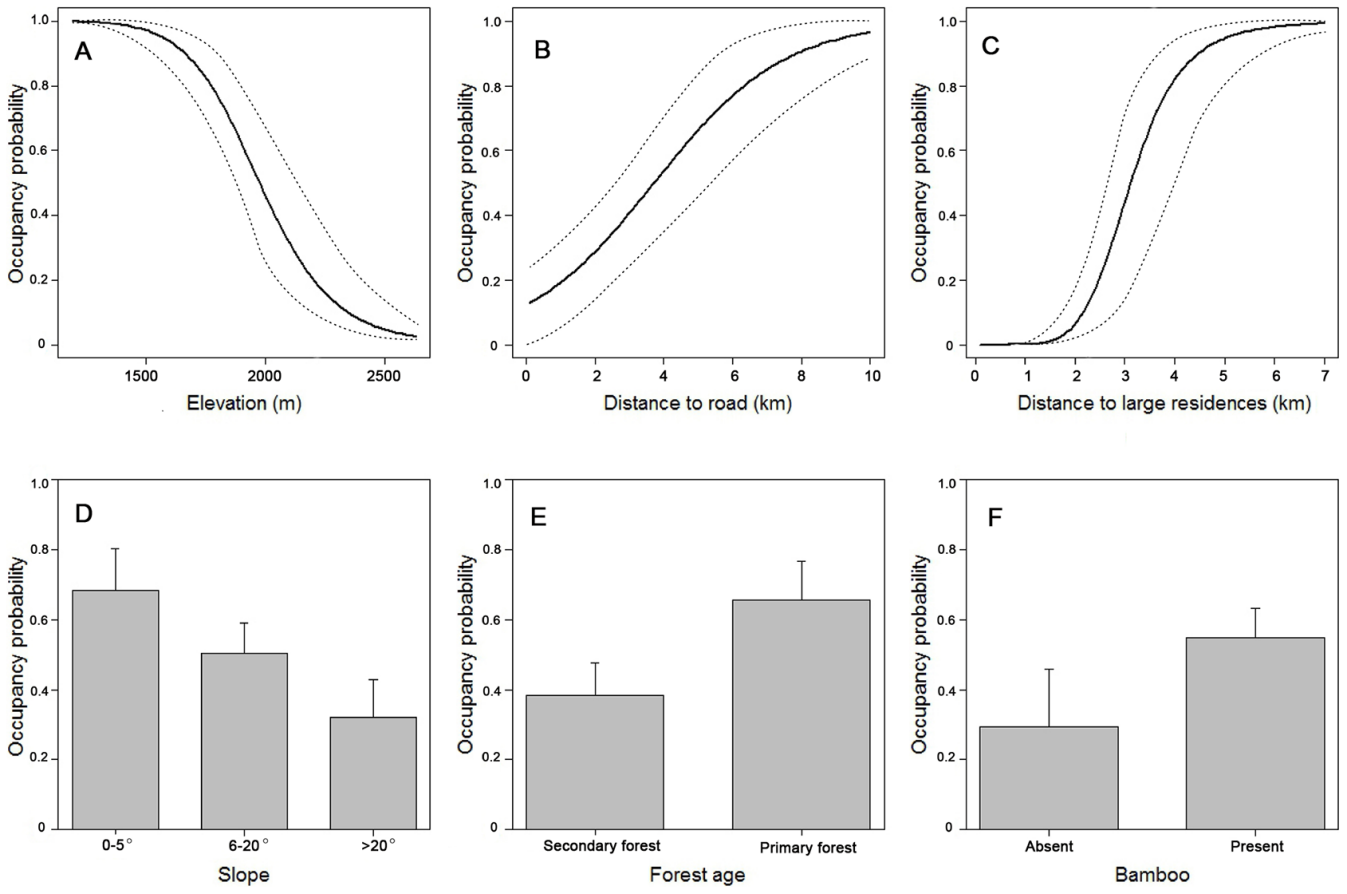
Partial correlation between giant panda occupancy probability and each environmental variable. The model-averaged weights is 1.00 for elevation (A), 0.46 to distance to road (B), 1.00 for distance to large residences (C), 0.30 for slope (D), 1.00 for forest age (E), and 0.45 for bamboo distribution (F).

Four variables were excluded from later analyses because of their high collinearity with other covariates (Table 1). Detection probability was high when scent lure was applied or temperature was low, but was not influenced by camera view. In the selection of occupancy covariates with scent lure and temperature as detection covariates, 13 models with Δ AICs <2 were identified as top models (Table 2). All the nine occupancy covariates were included in the final model-averaged predictive equation, among which six (i.e. elevation, slope, forest age, bamboo presence, distance to road, and distance to large residences) had model weights >0.3. Partial response curves suggested that the occupancy probabilities of giant pandas were higher in areas with lower elevation, less steep slope, and the presence of bamboo and/or primary forest (Fig. 2). In addition, being close to large residences or roads reduced the occupancy probabilities of giant pandas.

**Table 2:** Top models for predicting the occupancy probability of giant panda.

Occupancy covariates	number of parameters	AIC	ΔAIC	AIC weight	Cumulative weight
Ele, Ftp, Dtl	5	567.16	0	0.14	0.14
Ele, Ftp, Bam, Dtl	6	567.27	0.11	0.13	0.27
Ele, Slo, Ftp, Bam, Dtl	7	567.97	0.81	0.09	0.36
Ele, Slo, Ftp, Dtl	6	568.22	1.07	0.08	0.44
Ele, Ftp, Dtr, Dtl	6	568.33	1.18	0.08	0.52
Ele, Ftp, Dtl, Dts	6	568.67	1.51	0.07	0.58
Ele, Ftp, Bam, Dtr, Dtl	7	568.72	1.56	0.06	0.65
Ele, Ftp, Bam, Ntr, Dtl	7	568.79	1.63	0.06	0.71
Ele, Slo, Ftp, Bam, Dtr, Dtl	8	568.79	1.64	0.06	0.77
Ele, Age, Ftp, Dtl	6	568.80	1.64	0.06	0.83
Ele, Slo, Ftp, Dtr, Dtl	7	568.82	1.66	0.06	0.89
Ele, Ftp, Ntr, Dtl	6	568.95	1.799	0.06	0.95
Ele, Ftp, Bam, Dtl, Dts	7	569.03	1.88	0.05	1.00

Every model contains the same detection covariates (i.e. temperature and scent lure application).

*Ele: elevation; Ftp: forest composition; Dtl: distance to large residence; Bam: bamboo distribution; Slo: slope; Dtr: distance to road; Ntr: type of nature reserve; Age: forest age; Dts: distance to small residence.

**Figure 3:**
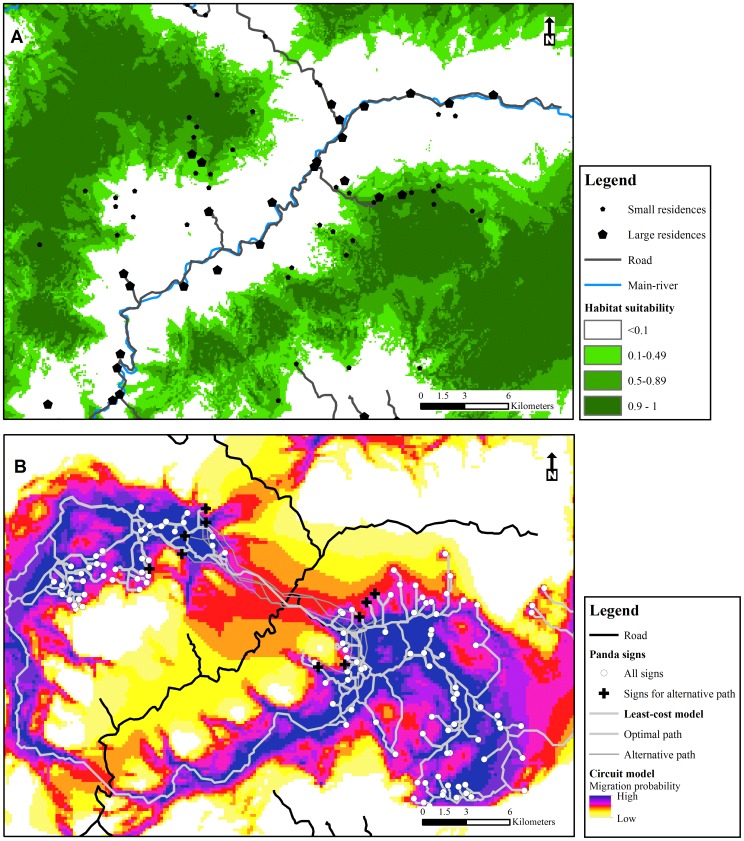
Maps for habitat connectivity and potential corridors. Habitat connectivity (A) predicted from occupancy model, and potential corridors (B) predicted by least-cost and circuit model.

Using the model-averaged predictive equation, we mapped the habitat suitability for the entire study area (Fig. 3a). The landscape had a broad fragmentation pattern: areas with predicted habitat suitability >0.5 (52% of the entire area) were mainly located further than 4 km from the XRV (55% covered by nature reserves), and areas with predicted habitat suitability <0.5 were mainly distributed near residences and roads along XRV. When comparing our prediction to the independent dataset collected by the SFD, the AUC value of 0.96 indicated strong predictive ability of our model.

### Potential Corridors

The least-cost model predicted two potential movement corridors (Fig. 3b). The length of the northern corridor was 17 km, and was located directly in the XRV. The length of the southern corridor was 40 km, and followed the forest along the mountain ridge before crossing the main road in the south. The alternative pathways identified remain relatively consistent with the optimal northern corridor predicted by the least-cost model (Fig. 3b).

The circuit model predicted the habitat connectivity pattern for the entire XRV Corridor area. It predicted two potential corridors similar to the corridors predicted by least-cost models but provided additional information (Fig. 3b). The width of the northern corridor was approximate 2 km, and the width of the southern corridor was approximate 1 km. Both corridors traverse a wide extent of low probability habitat along the XRV. The circuit model also predicted a third potential pathway using the Taibai Nature Reserve to the north of the proposed XRV Corridor with a length of approximately 35 km.

### Effectiveness of Alternative managements

The movement cost for optimal and alternative pathways were significantly different among scenarios (two-way-ANOVA, *F*_50, 1_ = 23.29, *p*<0.001). The dispersal cost under forest/bamboo restoration (X = 6453+634 SE) and the automobile tunnel construction scenario (X = 5843+629 SE) were significantly lower than dispersal cost in the current corridor (X = 8613+829 SE; paired *t*-test: forest/bamboo restoration: *t* = 10.34, *p*_holm_<0.001; automobile tunnel construction: *t* = 13.31, *p*_holm_<0.001), while dispersal cost under the residence relocation scenario was not significantly lower than the current corridor condition (X = 8509+827, *t* = 0.44, *p*_holm_ = 1). The automobile tunnel scenario resulted in lower dispersal cost than the forest/bamboo restoration scenario (*t* = 3.41, *p*_holm_<0.001).

## Discussion

We present a framework for using ground-based surveys to complement landscape attributes to examine large mammal habitat use across human-modified landscapes, and predict giant panda movement corridors. We based our corridor planning on predictions derived from this habitat model, a procedure widely used for conservation or land use planning [41]. Our study revealed that both natural and anthropogenic factors affect the distribution of giant pandas in the western Qinling Mountains. In particular, human infrastructures such as large residences and roads have strong negative effects and likely fragment giant panda habitat. Such results are consistent with previous studies. A negative relationship between giant panda distribution and the presence of roads and small villages was observed in adjacent areas in the Qinling Mountains [17]. Further south in the Qionglai Mountains, Li et al. [11] mapped movement corridors in Wolong Nature Reserve, and showed that a provincial highway had narrowed the movement pathway of giant pandas. Human land-use and habitat fragmentation have led to the drastic reduction of the most southern giant panda populations in the Xiaoxianling Mountains according to the genetic structure of the subpopulations [7]. Our studies did not include information on genetic structure, but our ground surveys across the XRV Corridor area found no evidence of movement between the established giant panda reserves.

### Giant panda habitat selection and distribution

Studies of other large mammals across diverse ecosystems have described a broad spectrum of negative effects of roads and other human infrastructures on wildlife habitat connectivity, both direct and indirect [42]. Major roads and urbanized areas are acting as barriers for carnivore species in Poland [43], and the attributes of roads (i.e. traffic volume and road type) and housing development were correlated with the number of deer-vehicle collisions in Virginia, United States [44]. Ungulate-vehicle accidents accounted for approximately 60% of the total police-reported traffic accidents in Sweden during the 1990s, and fences along roadsides limited animal movement and access to important resources [45]. As human infrastructure is so important in regulating animal movement, it should be considered in conservation planning for most large mammals.

In contrast to large residences and roads, giant pandas were detected near small residences. This finding opens the possibility of limits on household size being a viable management option to improve the corridor area. Household limits, and not outright housing bans, is contrary to studies suggesting intensive control of small residences within linkage areas [17]. Giant pandas and humans have co-existed within the XRV Corridor and the surrounding areas for centuries, as traditional agricultural activities only weakly infiltrated giant panda habitat [15]. Only within the past 50 years has intensive forest harvest and road construction, in combination with the government policy to aggregate families into larger residences along the river valley, altered the landscape such that it might limit giant panda movements across the valley [15], [16].

In determining the distribution of giant pandas across the XRV Corridor, we found that giant pandas used low elevation sites, in contrast with other studies where giant pandas selected higher-elevation areas [18], [46]. Avoidance of lower elevations would make a valley corridor, such as the XRV area, less permeable for giant pandas. The differences between our study and previous efforts might be that our low-elevation areas were not all modified by human infrastructure (i.e. large residences and roads). The paradigm of giant pandas being restricted to high elevations should be reexamined, with more comprehensive investigation on anthropogenic interferences to movement across valley areas.

Interesting to us was the lack of gradation in habitat occupancy, as most habitat was either highly suitable or unsuitable for giant pandas. This result may be attributed to giant panda's avoidance to large residences along the XRV (Fig. 2C). Such infrastructure avoidance created a distinctive barrier between nature reserves along the XRV, and probably limits movement between reserve populations. In order to improve the conservation status of giant pandas, the Chinese government has focused on nature reserve creation, and established more than 50 nature reserves in the past 40 years [14]. While populations have significantly increased inside their reserves, much of the landscapes between nature reserves lack habitat protection or land-use restrictions. For the present reserve system to be viable, giant pandas must be able to move among reserves across the matrix of anthropogenic features [7], [47]. In the Qinling Mountains, future conservation activities should reinforce the protection efforts at lower elevations and improve habitat linkages among core habitat areas [17]. Within the XRV, partial response curves indicate there are barriers to movement, and habitat connectivity can only be restored by removal of those barriers.

### Potential use of proposed corridor

Although we found no evidence of a movement corridor across the XRV Corridor area, this area still has potential to provide for movement based on the results of both our least-cost and circuit models. We produced a cost-surface map for giant panda movement, and identified potential movement corridors connecting two giant panda populations. All the potential corridors went through the same 2 km wide section of the XRV. The section has no large residences or cropland, but abundant bamboo distribution, and thus is the best candidate for future corridor establishment efforts.

There is one caveat to our analysis. Our modelling process was limited by our reliance on detection records to determine giant panda habitat use. Using movement data from radio-collared fishers (*Martes pennanti*) to test model predictions, LaPoint et al. [48] found that least-cost and circuit models were more accurate if movement data were incorporated in the model creation. Although some telemetry studies are currently being conducted with giant pandas [15], [49], these are confined to animals within reserves and do not provide movement information across the agricultural landscape between reserves. Our modelling process would be improved with telemetry data, but remains valid due to our ability to detect the animals within the agricultural matrix. In this case, field teams deploying camera-traps in association with the sign transects generated sufficient data for the modelling process, and model validation indicated a strong predictive ability.

### Conservation implications

To reveal potential options for land management to improve the XRV Corridor, we used a scenario approach to explore the future consequences of conservation decisions [50]. Instead of proposing a single-best solution, our scenario approach acknowledges the uncertainty inherent in the XRV Corridor by comparing the effectiveness of alternative future management options [51]. By contrasting alternative future states into a decision-making framework, the scenario approach allows scientists to work together with decision-makers, land managers and the public to explore the long-term consequences for conservation alternates [52]. We strongly fell this is the best approach to engaging the Chinese government in discussion of the XRV Corridor area.

One of the restoration activities examined was reforestation and bamboo planting, which is considered the priority management action for giant panda habitat restoration as well as corridor establishment in several regions including the Qinling Mountains [12], [13], [37]. According to our models, such management actions will increase available habitat since mature forest and bamboo understory are key factors to giant panda habitat use. Such results are consistent with study from larger scale, that pandas are associated with old-growth forest [37]. However, mature forest restoration is a long-term process whose success can be slowed by multiple factors [53], and habitat restoration does not remove the impermeable barriers created by roads and large residences. Therefore, forest restoration should continue, but does not meet the urgent current need of giant panda conservation, given that the XRV Corridor area is experiencing growth of human infrastructures that will supersede any forest improvements.

The Natural Forest Conservation Program (NFCP), a new forest policy has been adopted in China, advocates residence relocation as a major means of forest ecosystem restoration [54]. Though such initiatives have created better living conditions for some communities [55], its ecological consequences remained unpredictable [56], [57]. According to our scenario models, residence relocation could not meet the need to restore the habitat connectivity for giant panda in the XRV Corridor.

Our study suggests that the construction of an automobile tunnel may be the most effective approach in XRV corridor establishment, as this activity will simultaneously reduce the barriers of transportation and human residences. In this region, an automobile tunnel would divert automobile and truck transportation under the primary movement corridor. As a result, the original winding mountain highway will be abandoned and has the potential to function as a wildlife corridor. Among the 13 road structures (e.g. automobile tunnel, bridge, etc.) examined, automobile tunnels were the most effective at reducing vehicle-large ungulates collisions in the United States [58]. In the Qinling Mountains, automobile tunnel construction requires extensive landscape modification, and was found to displace goral (*Naemorhedus griseus*), serow (*Capricornis milneedwardsii*), and leopard (*Panthera pardus*) at a Qinling Mountain site [40]. However, five years after the automobile tunnel completion, giant pandas sign (footprints, dung, etc.) were found along the abandoned mountain highway, as the traffic had disappeared and the forest structure had partially recovered [59]. The lack of suitable habitat around the current highway indicates that any automobile tunnel construction would not reduce current panda habitat, but only benefit long-term conservation goals.

The automobile tunnel construction costs are usually high [58], but tunnels are increasingly used by the national and provincial governments to facilitate transportation across the Qinling Mountains [60]. In the past 10 years, more than 20 automobile tunnels were completed in Qinling Mountains, among which 5 were located in nature reserves, and 12 were located within 5 km of a nature reserves [60]. The newly planned Xian-Chengdu high-speed railroad calls for several additional tunnels in the region. While highways and high-speed rails construction usually create barriers for mammal movement, automobile tunnels within the landscape between giant panda reserves may provide opportunities to facilitate habitat connectivity by functioning as wildlife overpasses. The conservation benefits of these automobile tunnels have not been explored for giant pandas, but our models indicate they may be the best means to remove current barriers to giant panda movements.

## Supporting Information

Table S1 **Camera trapping data.** Camera trapping data collected during 2010 to 2013. 243 camera trap deployments have been set up with a total effort of 9136 camera nights.
(XLSX)

Table S2 **Bamboo dataset surveyed by the SFD.** The distribution of bamboo across the study area surveyed by the Shaanxi Forestry Department (SFD) during 2007 to 2010.
(XLSX)

Table S3 **Sign transect dataset surveyed by the SFD.** The sign transect dataset that was collected by the Shanxi Forestry Department (SFD) was used to validate our occupancy model.
(XLSX)

Table S4 **Giant panda distribution data.** Giant panda distribution data that was generated by combining the sign plot records with camera trap data.
(XLSX)
